# Postpartum delirious mania in a patient with diagnosis of bipolar disorder after cesarean delivery: a case report

**DOI:** 10.3389/fpsyt.2026.1821115

**Published:** 2026-06-10

**Authors:** Bowen Song, Yiwen Yuan, Zhixiong Li, Zhongrui Ma, Xiufang Yang, Zhe Li

**Affiliations:** 1 Mental Health Center of West China Hospital, Sichuan University, Chengdu, China; 2 National Center for Mental Disorders, West China Hospital, Sichuan University, Chengdu, China; 3 Department of Psychosomatic Medicine, Chengdu Fifth People’s Hospital, Chengdu, China; 4 The Third Department of Clinical Psychology, Karamay Hospital of Integrated Traditional Chinese and Western Medicine, Karamay, China

**Keywords:** bipolar disorder, case report, delirious mania, electroconvulsive therapy, postpartum psychosis

## Abstract

**Background:**

Postpartum depressive episodes are common. Patients with bipolar disorder (BD) may also experience manic or mixed episodes postpartum, but cases of delirious mania (DM)—characterized by rapidly evolving delirium, mania, and may involve psychotic symptoms—are rarely reported. Given its severity, frequent underdiagnosis, and life-threatening risks, a thorough exploration of its clinical features, potential risk factors, and treatment strategies can aid clinicians in early recognition and intervention, thereby improving patient outcomes.

**Case presentation:**

We report a rare case of postpartum delirium with manic features. The patient was a 27-year-old woman with a history of bipolar disorder type I. She underwent amniocentesis at 19 weeks of her first pregnancy and developed a manic episode with psychotic symptoms on the third postoperative day. She improved rapidly after receiving antipsychotic and sedative medications. This presentation occurred on day 2 following her second cesarean section. She exhibited a series of agitated symptoms including motor restlessness, verbose speech, auditory hallucinations, mood swings, irritability, aggressive behavior, and abnormal excitement. She also demonstrated delirious mania features such as disorientation in time and place, inattention, memory impairment, and urinary incontinence. Initial emergency department treatment with sodium valproate, quetiapine, and propofol proved ineffective. After admission, treatment was switched to sodium valproate and olanzapine, yet outcomes remained suboptimal. However, following electroconvulsive therapy (ECT), symptoms rapidly improved. During regular follow-up visits over 6 months post-discharge, the patient remained free of manic or delirious symptoms while continuing olanzapine and sodium valproate therapy, with overall satisfactory outcomes.

**Conclusion:**

Women with bipolar disorder and multiple childbirths may have an increased risk of delirious manic episodes. Electroconvulsive therapy (ECT) is an effective treatment option for rapid relief. Valproate and olanzapine can serve as maintenance therapy regimens.

## Background

The incidence of confusion and excessive self-blame is significantly higher in postpartum manic episodes than in non-postpartum episodes. Compared to non-postpartum episodes, postpartum episodes exhibit markedly fewer manic symptoms and significantly more depressive symptoms (1).Delirious mania is characterized by acute onset and rapid progression of symptoms, with varying course development. It primarily manifests in the following symptom categories: delirium symptoms (such as disorientation, altered perception, and abnormal mental status); manic symptoms (such as hyperactivity, insomnia, psychomotor agitation, behavioral disorganization, and impulsivity); psychotic symptoms (e.g., hallucinations or delusions); and catatonic symptoms (e.g., echolalia, stereotyped movements, postural rigidity, catatonic stupor, waxy flexibility, and fixed staring). It is a rare neuropsychiatric disorder (2).Current research on delirious mania is severely lacking (3), Therefore, the diagnosis and treatment of this disorder remains challenging.

Regarding the risk of severe manic episodes in women with bipolar I disorder, the risk of an episode during pregnancy is 5.2%, while the risk of a postpartum episode is 30.1%, with the highest risk occurring in the early postpartum period. The highest risk of relapse occurs after a live birth (34.4%), while the risk is lower following miscarriage (15.2%) and induced abortion (27.8%). Compared to women with uncomplicated first pregnancies, those experiencing complications during pregnancy or postpartum are less likely to have a second child. If they do have a second child, their relapse risk increases significantly with subsequent pregnancies (4).This finding suggests that women with bipolar I disorder who have experienced perinatal affective episodes may have an increased risk of relapse in subsequent pregnancies.

Although not included in the Diagnostic and Statistical Manual of Mental Disorders, 5th Edition (DSM-5) or the International Classification of Diseases, 11th Revision (ICD-11), this condition has been extensively documented in the historical literature (5).The etiology of delirious mania remains unclear. Previous studies suggest this syndrome may be associated with drug toxicity (steroids, stimulants, hallucinogens, psychotropic medications), metabolic disorders (azotemia, hepatotoxicity, lupus), and infectious diseases of the central nervous system. Women, younger patients, and individuals with bipolar disorder may be identified as high-risk groups for delirious mania (6).ECT is considered the most effective treatment for delirious mania based on case reports and series, with a high response rate. When ECT is not feasible, high-dose benzodiazepines should be administered (7).Recent reports indicate that delirious mania may account for up to 15% of all acute manic episodes. When delirious mania goes unrecognized or is inadequately treated, its severity can rapidly escalate and become life-threatening (8).Research indicates that individuals with bipolar disorder (BD) may experience an increased risk of manic or mixed episodes following childbirth (9).Women with bipolar disorder are at high risk of relapses during pregnancy and postpartum. Multiple pregnancies may be risk factors for postpartum psychosis and manic episodes (10).However, postpartum delirious mania cases are rarely reported, and no studies have suggested a relationship between multiple births and disease recurrence. Moreover, cases of delirious mania occurring postpartum are extremely rare. This patient developed delirious mania following a cesarean section during her second pregnancy, after having experienced a manic episode during her first pregnancy. Therefore, we report this case to draw the attention of clinicians.

## Case presentation

### Medical history

We report a 27-year-old Chinese female patient diagnosed with Bipolar Disorder Type I. In 2015, she began exhibiting symptoms of agitation and pressured speech, logorrhea and thought process changes, frequently calling family members late at night for hours on end. She spoke incessantly and was difficult to interrupt, using exaggerated language. She also developed compulsive shopping and spent money recklessly. These symptoms persisted for approximately two months before resolving spontaneously. In 2018, she again exhibited irritability and verbosity, engaging in prolonged conversations, constantly calling friends on her phone, frequently texting family members, becoming engrossed in reading books, using exaggerated language, experiencing heightened shopping urges, making impulsive purchases, repeatedly getting up at night to perform household chores, constantly checking whether her parents were asleep, and experiencing reduced sleep needs, sometimes sleeping only 2-3 hours. The patient was hospitalized at our institution with a diagnosis of “Bipolar Disorder Type I, currently in a manic episode without psychotic symptoms.” She received treatment with sodium valproate 1500mg/day and quetiapine 0.8g/day, remaining hospitalized for 17 days. After emotional symptoms improved, medication was continued for 6 months before she ultimately discontinued it on her own. During routine prenatal amniocentesis at 19 weeks of her first pregnancy in 2020, manic symptoms recurred, including excitement, insomnia, contradictory hallucinations, delusions of reference, and abnormal behaviors such as talking to herself and repeatedly getting up. She was readmitted with a diagnosis of “Bipolar Disorder Type I, manic episode with psychotic symptoms.” She received treatment with “sodium valproate 1500 mg/day and quetiapine 0.7 g/day” and was discharged after a 15-day hospitalization. Six months post-discharge, she discontinued sodium valproate on her own but continued long-term quetiapine at 0.2 g/day. Her first child was delivered via cesarean section in 2021. Postpartum, her mood remained stable while maintaining a quetiapine dose of 0.1 g/day.

In January 2024, she conceived her second child. Throughout pregnancy, she maintained daily quetiapine 0.2 g dosing with stable mood. In October 2024, she delivered a son via cesarean section. However, on the first postoperative day, she abruptly developed symptoms including excitement, excessive talking, agitated movements, and heightened shopping urges. She exhibited severe mood swings, irritability, and even aggression toward family members. Her sleep requirement markedly decreased, resulting in three consecutive days of insomnia. She experienced auditory hallucinations, hearing people mocking her downstairs, accompanied by paranoid delusions that her husband had been unfaithful during the bridesmaid ceremony. She exhibited inappropriate toileting behavior and bedwetting. Her mental state became disorganized, claiming to have lived for five or six hundred years and stating she was teaching and educating people while at home. She became confused, unable to recognize time, people, or places.

The patient had no previous chronic physical diseases, no significant genetic history in the family, and generally had good social support.

The patient was brought to our emergency department by family members. The patient was unconscious, extremely agitated and restless, and uncooperative during physical examination. Vital signs: T: 38.2°C, P: 102/min, R: 18/min, BP: 106/73 mmHg. Complete blood count showed hemoglobin 109 g/L (reference range 115-150 g/L), with normal white blood cell count. Electrolyte and liver function tests were normal. Thyroid function showed no abnormalities. D-dimer was 1.97 mg/L FEU (reference range <0.55 mg/L FEU).

Emergency treatment included quetiapine 200 mg/day and sodium valproate 500 mg/day. After 2 days of treatment, manic symptoms showed no significant improvement. The patient exhibited impulsive behavior, disorganized speech and actions, accompanied by aggressive behavior. An intramuscular injection of 10mg haloperidol was administered with poor response. Protective restraints were applied and an indwelling urinary catheter was inserted. On day 3, continuous propofol infusion at 5mL/hour was initiated for sedation. Due to extreme agitation posing a risk to herself and staff, she required sedation with a continuous propofol infusion in the emergency setting. By the fourth day, symptoms persisted, prompting referral to our department. On admission, the patient exhibited mixed delirium and manic symptoms, demonstrating inability to orient to time, place, or person. Despite continuous restraint, the patient repeatedly sat up at night, exhibiting agitation, incoherent speech, and intermittent symptoms including restlessness, incessant talking, drowsiness, recent memory impairment, and echolalia. Physical examination revealed normal blood pressure (117/83 mmHg), respiratory rate (20 breaths/min), and heart rate (89 beats/min). Neurological examination showed no positive findings.

Head/chest/abdominal CT scans demonstrated no significant abnormalities in brain parenchyma, with mild bladder distension. All other findings were unremarkable. Standard echocardiography and electrocardiogram showed no significant abnormalities. Complete blood count revealed hemoglobin at 99 g/L (reference range 115-150 g/L). Electrolyte and liver function tests were normal. Thyroid function was unremarkable. Inflammatory markers including white blood cell count, procalcitonin, interleukin-6, and C-reactive protein showed no significant abnormalities. Urinalysis: - Hematuria: 50 (2+) per μL - Protein: Qualitative 1.0 (2+) g/L - Urobilinogen: Qualitative 68 (2+) μmol/L - Red blood cells: 56/μL - Red blood cells (high-power field): 10/HP Drug screening for uremic toxins: Negative. DRS-R-98(Delirium Rating Scale, DRS-R-98):The total score for the severity items of this patient was 36 points (0-39 points), the total score for the diagnostic items was 4 points (0-6 points), and the total score range was 40 points, indicating the presence of severe delirium.YMRS(Young Mania Rating Scale,YMRS):30points,indicating the presence of severe mania. Following consultation with Neurology, cerebrospinal fluid analysis was recommended but declined by the guardian.

### Diagnosis relevant

This episode occurred on the second day post-cesarean section, presenting with acute onset and severe symptoms. We initially considered acute manic episode, but based on the patient’s presentation, head imaging, and neurological examination, no significant positive findings were identified. Although a lumbar puncture was recommended to definitively rule out autoimmune encephalitis and other conditions, the family declined, which represents the primary limitation of our diagnostic efforts. Ultimately, based on the patient’s established history of type I bipolar disorder, the acute stressor (cesarean section), and exclusion of other common causes, we consider her clinical presentation most consistent with a rare, severe postpartum psychotic episode—delirious mania with psychotic features.

### Treatment

Upon admission, treatment was adjusted to olanzapine (5 mg daily) and sodium valproate (1000 mg daily). Initial blood tests revealed mild anemia, and urine analysis showed positive occult blood. The patient reported no symptoms of urinary frequency, urgency, or dysuria. Following consultation with an internist, symptomatic hemostasis was initiated with carbazochrome sodium sultanate, supplemented by intravenous fluid replacement to maintain fluid balance.

On the first day after admission, the patient remained in a state of confusion, unable to recognize persons, places, or time. She exhibited daytime muttering with incoherent speech, unprovoked shouting and laughing, and marked emotional fluctuations. Olanzapine dosage was gradually titrated to 20 mg/day. Despite the increase in olanzapine to 20 mg/day, the patient’s clinical condition did not improve. She continued to exhibit a severe and fluctuating course of confusion, psychomotor agitation, and psychosis. We concluded that delirious mania was highly probable and treatment would be guided accordingly.

On the seventh day of hospitalization, following informed consent from the patient’s legal guardian, we initiated electroconvulsive therapy (ECT). (ECT machine: Thymatron^®^;Electrode Placement : Bitemporal,BT;Electrical stimulation intensity:1.5-2 seizure threshold,ST).We planned to administer ECT daily for the first three consecutive days. For subsequent treatment, we delivered it every other day, with frequency adjusted according to the patient’s actual condition. Treatment protocols maintained olanzapine at 20 mg/day and sodium valproate at 1000 mg/day. Following three consecutive treatments, the patient’s mood gradually stabilized, with markedly improved consciousness clarity enabling recognition of time and individuals. When asked about her husband’s hospitalization, she reported no recollection of related events since delivery and no recurrence of impulsive aggressive behavior. Given that sodium valproate may interfere with ECT efficacy, we adjusted its dosage to 500 mg/day. Concurrently, blood tests revealed hemoglobin at 106 g/L (reference range: 115-150 g/L), while urine analysis showed no significant abnormalities, allowing discontinuation of Carsulfonate Sodium.

Following the sixth ECT session, the patient’s mood stabilized. She was frequently observed walking with her husband in the ward, speaking fluently and interacting amicably with others. Although occasional self-talk persisted, her orientation had fully recovered, amnesia symptoms resolved, and significant hallucinations and delusions subsided. After the eighth treatment, her emotional state became more consistent, with no recurrence of hallucinations, delusions, agitation, or excessive talkativeness. She felt calmer, no longer hearing mocking voices or experiencing delusions about her husband’s infidelity. She expressed gratitude for her husband’s companionship and longed for her newborn child at home. She was discharged on the 19th day of hospitalization and continued daily medication with 20mg of olanzapine and 500mg of sodium valproate post-discharge.

### Follow-up

**Figure 1:**
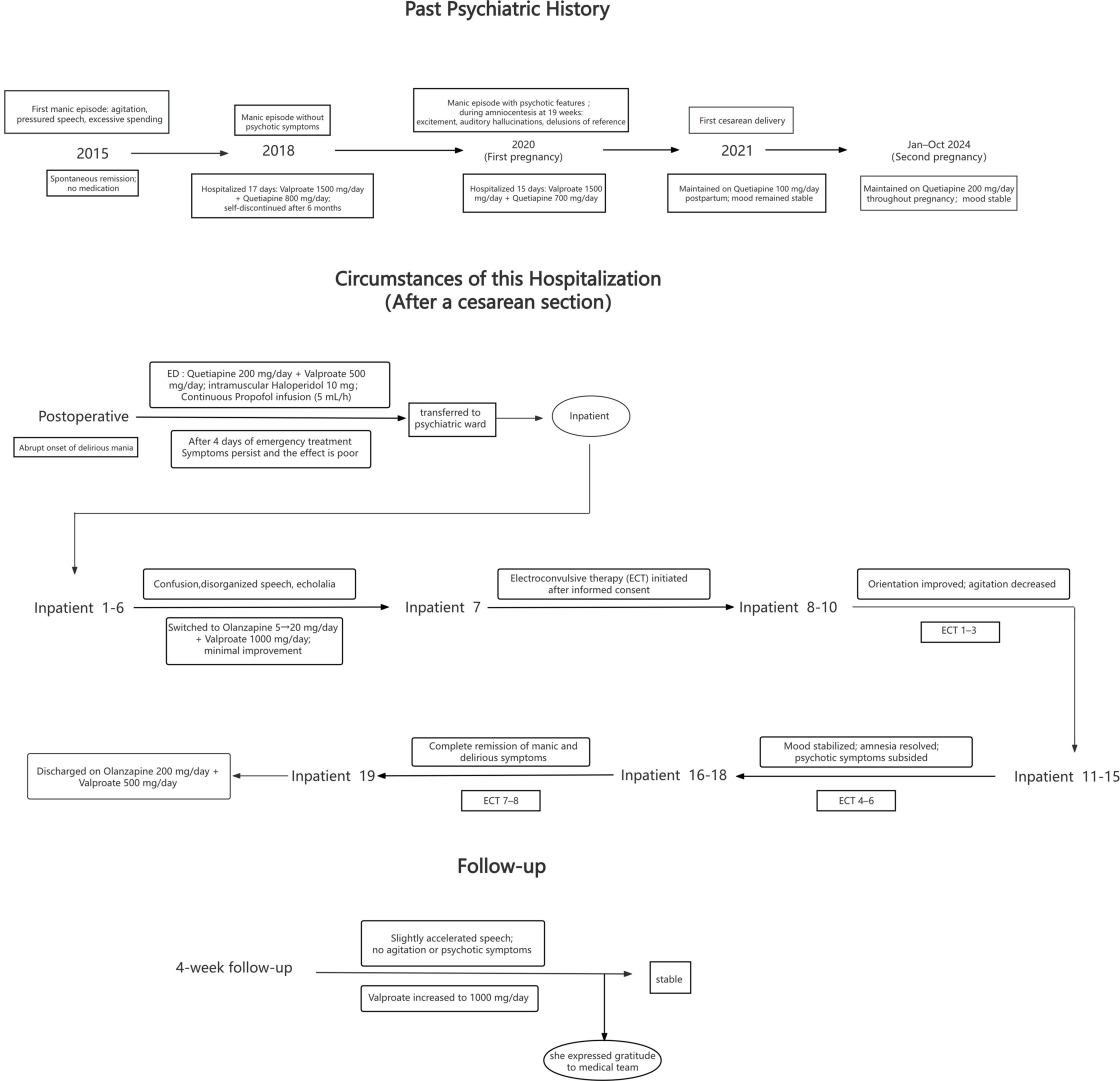
Clinical treatment timeline and treatment process.

At the 4-week post-discharge follow-up, the patient was alert and oriented, with slightly accelerated speech pace but no signs of agitation or hyperactivity. Impulsive aggression was absent, and sleep requirements remained unchanged. Her husband reported that 2 weeks after discharge, he observed accelerated thought processes and slightly faster speech, but overall emotional stability persisted. Subsequent outpatient adjustments modified the medication regimen to olanzapine 20 mg/day and sodium valproate 1000 mg/day. The patient reported complete amnesia for the acute phase, stating, “I don’t remember anything from the delivery until around the third ECT treatment—it’s just a blank.” And she expressed gratitude to medical team. Clinical treatment timeline and treatment process are shown in **Figure 1**.

## Discussion

Delirious mania is a condition characterized by the simultaneous occurrence of delirium and mania. Little is known about its etiology and psychopathology, with multiple hypotheses currently proposed, such as dysregulation of the dopamine transporter system (11), abnormal serotonin levels (12), altered Gamma-Aminobutyric Acid (GABA) transmission (13), even COVID-19 can potentially lead to Delirious mania (14).It should be noted that most patients have underlying systemic or psychiatric conditions, with those suffering from bipolar disorder and schizoaffective disorder being particularly susceptible. Different studies show varying proportions of delirious mania among manic patients: one study indicates that approximately 19% of cases exhibit this symptom characteristic (15).Other studies, however, estimate the figure to be between 15% and 25% (16).In 2021, Elias and colleagues calculated that approximately 15% of manic patients die from overexertion, suggesting that these cases may involve misdiagnosed delirious mania (17).Research specifically addressing the incidence, specific risk factors, prevention, and treatment of postpartum delirious mania is currently severely lacking. Consequently, postpartum delirious mania carries a high risk of misdiagnosis and underdiagnosis (18, 19).

Although approximately 50% of women with postpartum psychosis had no prior history of psychiatric episodes, there is a significant association with bipolar disorder, particularly bipolar disorder type I (20).The birth of a child is a potent trigger for severe mental illness episodes, and while this risk is not uniformly distributed across all mental disorders, it is specifically associated with bipolar disorder (21).In our case, the patient experienced a manic episode during her first pregnancy, which was followed by a second pregnancy. This suggests that although no manic episode occurred after the first pregnancy, the risk of manic recurrence may increase with subsequent pregnancies. Investigating the risk of manic relapse with delirium in women with bipolar disorder after childbirth—particularly in those who have given birth multiple times—holds significant clinical value.

Young women are more susceptible to the psychological impacts of childbirth, particularly depression and anxiety. The effects of live birth on mental health are age-dependent, with older women facing a higher likelihood of developing postpartum psychosis (22).Moreover, from the perspective of obstetric delivery methods, emergency cesarean sections may be positively correlated with first-episode postpartum psychosis (23).Previous studies suggest that psychosocial factors, hormonal levels, immune function, circadian rhythms, and genetic factors may play significant roles in postpartum mental illness (6).Our patient is a 27-year-old woman with two prior cesarean sections. Although she did not exhibit significant manic symptoms after the first delivery, she experienced a manic episode during amniocentesis performed in her subsequent pregnancy. This suggests that not only postpartum factors but also physical and psychological stressors such as surgical procedures during pregnancy may influence the onset of manic episodes. Additionally, reproductive hormone levels (estrogen and progesterone) surge dramatically during pregnancy and then plummet immediately after childbirth (21).This may be related to our patient’s sudden onset of delirious mania following her second cesarean section.

Diagnosing delirious mania requires ruling out underlying systemic diseases. As with other psychiatric diagnoses, it necessitates rigorously excluding multiple causes such as medications, psychoactive substance use, electrolyte abnormalities, hormones, infections, and autoimmune disorders (e.g., anti-N-methyl-D-aspartate [NMDA] receptor encephalitis). Therefore, thorough medical examinations are essential, including MRI, lumbar puncture for neuronal antibody testing, and toxicology screening. Prior to diagnosing delirious mania, we had already completed comprehensive tests such as head MRI, blood parameter monitoring, and thyroid function tests to rule out possibilities caused by brain disorders, systemic inflammation, or thyroid dysfunction. Following consultation between neurology and our department, lumbar puncture was recommended to rule out organic causes. However, the patient’s legal guardian refused consent, leaving autoimmune encephalitis as a potential but unconfirmed possibility. Nevertheless, subsequent treatment efficacy indicates autoimmune encephalitis can be provisionally excluded based on our clinical management outcomes. The diagnosis remains provisional pending exclusion of other causes, with the family’s refusal of lumbar puncture being a significant barrier to definitive diagnosis.

Regarding treatment, particular caution should be exercised when selecting pharmacotherapy to avoid high-potency antipsychotics, as they may exacerbate extrapyramidal symptoms and induce seizures or neuroleptic malignant syndrome. Anticholinergic medications should also be avoided, as they may exacerbate confusion. Although lithium salts are effective for bipolar disorder, high-dose lithium in delirious mania may cause severe encephalopathy syndrome, presenting with symptoms such as somnolence, tremor, cerebellar dysfunction, and worsening confusion; it may also lead to extensive and irreversible brain damage (8) (24).Antipsychotic drugs and valproate are both effective for the treatment of acute mania (25).Given the suboptimal efficacy of quetiapine in our patient, we opted for valproate combined with olanzapine to control symptoms. We avoided benzodiazepines due to their risk of inducing or exacerbating delirium and excessive sedation (26).However, after switching treatment regimens for approximately six days, the results remained unsatisfactory. Previous studies suggest medication require longer periods to take effect, while ECT demonstrates significant efficacy in treating delirium and mania, with remission rates reaching 80% to 100% (27).Given the severity of the syndrome’s symptoms, the use of these medications alone cannot be considered an effective treatment option during the acute phase. Consequently, we subsequently initiated ECT treatment. The delirium began to stabilize after three consecutive ECT sessions and resolved after eight sessions. This suggests that in future clinical practice, initiating ECT immediately upon recognizing delirious mania may represent a superior clinical decision.

## Conclusion

Currently, no effective tools exist to identify prodromal or early symptoms of delirious mania occurring during pregnancy. Women with bipolar I disorder, particularly those who have experienced mania or psychosis during pregnancy, may face an increased risk of recurrent postpartum delirious manic episodes despite prophylactic mood stabilizer use after delivery. Therefore, women with bipolar disorder should undergo careful monitoring and follow-up during multiple pregnancies or postpartum periods to remain vigilant for the risk of postpartum delirious mania. Clinicians must be able to rapidly recognize the symptoms of this syndrome and initiate effective treatment to reduce its mortality rate.

## Data Availability

The original contributions presented in the study are included in the article/supplementary material. Further inquiries can be directed to the corresponding authors.
